# Apoptotic cell-linked immunoregulation: implications for promoting immune tolerance in transplantation

**DOI:** 10.1186/s13578-015-0019-9

**Published:** 2015-06-07

**Authors:** Ruixia Kuang, Sylvain Perruche, WanJun Chen

**Affiliations:** Department of Plastic Surgery, The Affiliated Hospital of Qingdao University, Qingdao, China; Inserm, UMR1098, Besançon, France; Mucosal Immunology Section, OPCB,NIDCR, NIH, Bethesda, MD 20892 USA

## Abstract

The induction of alloantigen-specific immune tolerance is the “Holy-Grail” in transplantation. Although it had been previously demonstrated that transient depletion of T cells through apoptosis could lead to long-term immune tolerance, the underlying mechanism responsible for this tolerance induction and maintenance was unknown. In this short article, a novel mechanism for long-term immune tolerance via transient T cell apoptosis will be discussed, based on our recent findings in a CD3-specific antibody treatment-induced immune tolerance mouse model. Transforming growth factor-β, which is produced by immature dendritic cells whilst they phagocytose apoptotic T cells and by macrophages, plays an important role in initiating long-term immune tolerance. A possible model of how allospecific-immune tolerance can be induced in order to prevent allograft rejection in transplantation will be also proposed.

## Introduction

Despite the majority of the cells in the body being healthy and functional, there are still considerable numbers of cells dying through apoptosis every day. The death of a cell and the release of its contents could lead to tissue damage and is associated with the risk of development of inflammation and autoimmunity if the apoptotic/dead cells are left unchecked or uncleared. However, very few people develop symptoms of autoimmunity and/or chronic inflammation. The most plausible explanation to this mystery is that apoptotic cells are rapidly and sufficiently engulfed and digested by phagocytes, such as macrophages. It has long been believed that this “passive” phagocytosis and clearance of apoptotic cells by macrophages was the sole mechanism that prevented the body from potential damage caused by the release of the contents of late apoptotic cells (when cell membrane of apoptotic cells is damaged). Although the idea of “passive phagocytosis” is an established fact in the biology of apoptosis, it may represent only the tip of the iceberg. Recent advances in our understanding of apoptosis show that “active suppression” may also be involved and this accounts for the “quiescent” and “immuno-unresponsive” status associated with the clearance of apoptotic cells. This concept of “active suppression” is supported by recent findings showing that macrophages produce and release the immunoregulatory cytokines transforming growth factor-beta (TGF-β) and interleukin IL-10 (IL-10) [1–4] when they contact, engulf, and digest apoptotic cells in cell culture. These immunoregulatory cytokines may in turn participate in preventing and/or suppressing the possible deleterious after-effects of cell death and of phagocytosis of apoptotic cells. Although this piece of in vitro evidence provided a new clue that helped us understand this “immuno-quiescence” phenomenon associated with apoptotic cell clearance, it was unknown whether this process occurs in vivo, and more importantly, if it is involved in the prevention and suppression of diseases such as transplant rejection and autoimmunity.

CD4^+^ CD25^+^ Foxp3^+^ regulatory cells (Treg cells) are instrumental in the induction and maintenance of peripheral immune tolerance [5–10] and of immune tolerance in transplantation [11–13]. Despite the fact that the majority of CD4^+^ Foxp3^+^ T cells are developed in the thymus as so called “natural” Treg cells, compelling evidence has suggests that TGF-β, in the context of T cell receptor engagement, induces Foxp3 expression from naïve CD4^+^ Foxp3– peripheral T cells and converts them into the regulatory phenotype known as adoptive Treg cells [14]. These adoptive Treg cells are functionally and phenotypically indistinguishable from the natural Treg cells. IL-2 is a critical cytokine in the induction and expansion of the adoptive Treg cells [15, 16]. The conversion to adoptive Treg cells also occurs in vivo, for which TGF-β signaling is required [17, 18]. Despite the general consensus that TGF-β is absolutely required for the Treg cell generation, it remains largely unknown what types of immune cells are the major source of TGF-β in vivo. Foxp3^+^ Treg cells themselves could be a cellular source of TGF-β [5, 19–21], which then “infectiously” converts naïve CD4+ T cells into adoptive Foxp3^+^ Treg cells [13, 22]. Indeed, macrophages [2, 23] and immature dendritic cells (iDCs) [24, 25] produce TGF-β. A critical question to be answered is how these phagocytes are triggered to release TGF-β in vivo and thereby consequently contributing to Treg cell generation and immune tolerance.

### Immune tolerance associated with T cell depletion

It is well established that transient T cell depletion (largely through apoptosis) induces long-term immune tolerance. A CD3-specific antibody (OKT3) was the first monoclonal antibody that was used as an immunosuppressive agent in clinical renal transplantation [26] and has been in use for almost three decades [27]. Monoclonal antibodies against murine CD3 (e.g. Clone 145-2c11) have also been used in several animal models of transplantation and autoimmune diseases [27, 28]. Monoclonal antibodies to both human and mouse CD3-specific antigen rapidly and efficiently deplete large numbers of T cells (about of 50 % of the T cell population) in the recipients, is largely via induction of apoptosis. Administration of a CD3-specific antibody injection resulted in short term immunosuppression followed by long-term immune tolerance, although the initial antibody injection induced a transient “flu-like” side effect [28].

Besides CD3-specific antibodies, there have been several other antibodies used to deplete T cells in order to induce immune tolerance. In 1989, Waldmann’s group showed that depletion of CD4^+^ T cells after exposure to certain antigens resulted in long-term specific immune tolerance in a study in mice [29]. Steinman and his colleagues treated mice with an antibody to CD4, which prevented the development of experimental autoimmune encephalomyelitis (EAE); in animals already paralyzed, administration of the same antibody reversed the EAE [30]. Recently, it was demonstrated in mice that selective depletion of alloantigen T effector cells via blockade of co-stimulatory molecules plus administration of rapamycin permitted acceptance of the allograft [31, 32]. The allograft was not rejected despite the complete recovery of peripheral T cells after the treatment was stopped. This raised an important question as to why the newly maturing T cells did not replace these deleted T cells and did not reject the graft or initiate a new wave of inflammation after cessation of the treatment [33]. Taken together, all these data suggest that there is a common underlying mechanism of immune tolerance that follows after apoptosis of T cells. The question is: how do apoptotic cells trigger the immune system to become tolerant, and what are the mediator(s) that link T cell apoptosis to long-term immune tolerance?

### The role of macrophage-derived TGF-β in apoptotic cell-mediated immune tolerance

We used CD3-specific antibody-induced tolerance in mice as a model to study the mechanisms of apoptotic cell-mediated immune tolerance. Based on the observations that a CD3-specific antibody injection induced T cell apoptosis [28] and that macrophages produce TGF-β after phagocytosis of apoptotic cells in cell culture [1, 2, 34], we hypothesized that the CD3-specific antibody might induce TGF-β production in vivo through clearing (phagocytosis) of apoptotic T cells. Indeed, intact CD3-specific antibody treatment increased total levels of systemic TGF-β in normal mice [34]. These elevated levels of systemic TGF-β can last for at least a few days. Notably, the kinetics of the increase in circulating TGF-β levels was different from that of inflammatory cytokines [28]. While the levels of circulating inflammatory cytokines, including tumor necrosis factor (TNF), interferon (IFN)-γ and IL-2, reached a peak at about 90 min and largely dropped off to almost zero by 6 h after the initial CD3-specific antibody injection [35], the peak in the total TGF-β in the circulation was not observed until about 24 h after the initial injection [34]. The difference in the kinetics between TGF-β and other inflammatory cytokines suggests that they may be derived from different cellular sources. Indeed, circulating TNF was produced exclusively by T cells following CD3- specific antibody injection [35]. Whereas TGF-β was mainly produced by phagocytes, particularly macrophages and immature dendritic cells [34] (discussed below), as demonstrated in a study where depletion of these phagocytes with clodronate-loaded liposome abrogated the increase in TGF-β in animals treated with a CD3-specific antibody. This production of TGF-β by macrophages is triggered largely by apoptotic T cells (in which apoptosis was induced by CD3-specific antibodies). Further supporting evidence comes from the observation that macrophages could very efficiently contact, engulf and ingest apoptotic T cells not only in cell culture, but also in vivo [34]. Direct examination of the macrophages ex vivo 24 h after the CD3-specific antibody injection, revealed that they expressed significantly higher levels of a membrane-bound latent-form of TGF-β (LAP-TGF-β) than untreated macrophages [34]. In addition, injection of mice with non-mitogenic CD3-antibodies (e.g. CD3-ImmunoglobulinG3) that caused substantially less T cell apoptosis failed to result in a significant increase in systemic TGF-β levels [36, 37]. The evidence collectively identifies macrophages as a key cellular source for the increase in TGF-β following CD3-specific antibody induction of T cell apoptosis.

### Immature dendritic cells in apoptotic cell-mediated immune tolerance

Notably, iDCs also produce TGF-β through the phagocytosis of apoptotic cells and thus contribute to apoptotic cell-linked immune tolerance. iDCs express lower levels of the co-stimulatory molecules CD80, CD86 and MHC II and do not express CD83 [38]. In contrast to mature DCs, iDCs are incapable of initiating effective Th1 and Th2 responses, but they can induce T cell tolerance. A unique feature of iDCs is their ability to engulf and digest apoptotic cells. Although previous studies emphasized an antigen-presenting function of iDCs after uptake of apoptotic cells, none examined whether the digestion of apoptotic cells affected their function of inducing adoptive immune responses. Recently, it has been reported that iDCs express TGF-β consequently resulting in the conversion of naïve CD4^+^ T cells into Foxp3^+^ Treg cells [25, 39, 40]. We found that exposure of iDCs to apoptotic cells substantially increased their TGF-β secretion and consequently strengthened their ability to convert naïve CD4^+^ T cells into Foxp3^+^ Treg cells [34]. This occurred in cell culture as well as in vivo; in mice, iDCs potently engulf apoptotic T cells and selective depletion of iDCs with clodronate-liposomes interferes with this increase in CD4^+^ Foxp3^+^ Treg cells induced by the CD3-specific antibody. Thus, it would be reasonable to envision that iDCs actively participate in the induction of CD4^+^ Foxp3^+^ Treg cells and immune tolerance because of the TGF-β they produce after ingestion of apoptotic cells.

### A link between apoptotic cells and the upregulation of CD4+ Foxp3+ Treg cells in vivo

The fact that both macrophages and iDCs produce TGF-β upon digestion of apoptotic cells and convert naïve CD4^+^ T cells into Foxp3^+^ Treg cells raises the possibility that this process may also influence the frequency of Foxp3^+^ Treg cells in vivo. Indeed, CD3- specific antibody treatment increases the frequency of Foxp3^+^ CD4^+^ Treg cells in mice. As early as 24 h after antibody injection, the relative percentage of Foxp3^+^ Treg cells in the CD4^+^ T cell compartment had already increased in the peripheral lymphoid tissues and blood [34]. It is unclear whether this immediate increase was associated with the relative resistance to death of natural Treg cells in response to a CD3-specific antibody. Intriguingly, by four to ten days and even one to two months after the CD3-antibody injection, the frequency of CD4^+^ Foxp3^+^ Treg cells is still substantially higher than that in the untreated mice (34 and unpublished data). This indicates that the increase in the number of Treg cells may also be attributable to a population converted from naïve CD4^+^ T cells, although a lymphopenic-driven expansion of existing T_reg_ cells caused by CD3-antibody treatment may also be involved. Notably, this increase in CD4^+^ Foxp3^+^ Treg cells is associated with the endogenous macrophages and iDCs, because depletion of these phagocytes abrogated the upregulation of Treg cells, accompanied by a decrease in TGF-β levels [34]. Therefore, it is conceivable that the apoptotic T cells trigger macrophages and iDCs to produce TGF-β, which in turn generates CD4^+^ Foxp3^+^ Treg cells. The increase in the ratio between Foxp3^+^ Treg cells and T effector cells is likely to be the major operating force in maintaining long-term immune tolerance.

### A critical function of phagocyte-derived TGF-β in the prevention and treatment of chronic inflammation

The apoptotic T cell-phagocyte that mediated immune tolerance was also tested and proven to be functional in the prevention and treatment of autoimmune diseases in an animal model of autoimmune disease - EAE. Firstly, treatment of mice with a CD3- specific antibody before the pathogenic peptide immunization effectively prevented the onset and development of EAE in myelin oligodendrocyte glycoprotein-induced EAE in C57BL/6 mice [34]. In this animal model, one might argue that the treated mice are still in a state of T cell deficiency four to five days after the CD3-specific antibody injection, and thus the immune unresponsiveness might be due to the lack of sufficient numbers of T cells. While this argument is reasonable, the mice with the same CD3-specific treatment plus phagocyte depletion had a similar number of T cells as the mice treated with the antibody alone, but exhibited a complete reversal of the suppression of EAE. A more striking effect was observed in the “treatment” experiment. The SJL mice were immunized with the proteolipid protein peptide PLP139-151 in the presence of complete freund adjuvant to establish EAE and were then treated with CD3-specific antibodies.

The CD3-specific antibody treatment led to a dramatic improvement of the disease course in this relapsing-remitting EAE, demonstrated by the delay of the first relapse, a decrease in the severity of the clinical score, and a reduction in the frequency of relapses [34]. The benefit of the CD3-specific antibody treatment was long lasting and was associated with an immunoregulatory dominant status in the mice. Evidence in support of this conclusion is the upregulation of the frequency of CD4^+^ Foxp3^+^ Treg cells in the spleen and in the target central nervous tissues (spinal cord), leading to an increase in the ratio between Treg cells to CD25^+^ Foxp3– CD4^+^ T effector cells or to Th17 or Th1 cells. Significantly, the therapeutic effect was completely reversed by pre-depletion of the phagocytes or neutralization of TGF-β with a blocking antibody before the CD3-specific antibody treatment.

### Immune tolerance induced by apoptotic cells with other regimens

In addition to antibody-mediated depletion of T cells, other regimens and means have been shown to induce long-term immune tolerance through induction of apoptosis. In this regard, it has been shown that an infusion of ethylene carbodiimide-treated donor splenic antigen-presenting cells results in indefinite survival of allogeneic islet grafts [41]. The mechanisms for ethylene carbodiimide-treated cell induced tolerance may involve the apoptosis-mediated TGF-β pathway, because ethylene carbodiimide induces the cells to undergo rapid apoptosis, and the tolerance can be completely abrogated by the neutralization of TGF-β or depletion of CD4^+^ CD25^+^ Foxp3^+^ Treg cells. Moreover, intravenous infusion of apoptotic splenic cells induced TGF-β-dependent Treg cell generation that in turn facilitated allogeneic bone marrow engraftment and delayed the onset of graft-versus-host disease [42]. Lastly, extracorporeal photochemotherapy (ECP) (a therapeutic approach used to treat severe graft-versus-host disease) generates significant numbers of apoptotic leukocytes [43] and is also known to induce Treg cells in vivo [44]. Whether apoptotic cell removal by phagocytes is involved in this generation of Treg cells remains to be determined.

Besides T cells, depletion of B cells or plasma cells also leads to the control of the deleterious immune response and this mechanism has been recently applied to the treatment of autoimmune diseases. Although these studies emphasized the depletion of certain type(s) of pathogenic B cells to achieve a therapeutic effect, it would not be impossible that the B cell depletion might also lead to similar apoptosis/phagocytosis‐mediated immunoregulatory mechanisms as described for T cell depletion. It would be interesting to investigate if this was the case because this would provide another option to achieve immune tolerance in transplantation. However, it should be pointed out that T cell receptor engagement is required to convert naïve T cells into Treg cells in addition to TGF-β secreted by phagocytes clearing apoptotic cells. Thus, whether B cell depletion could induce Treg cells may also depend on the T cell receptor engagement of the antigen-specific T cells.

### Manipulation of apoptotic cell-mediated immune tolerance: implications for transplantation

**Fig. 1:**
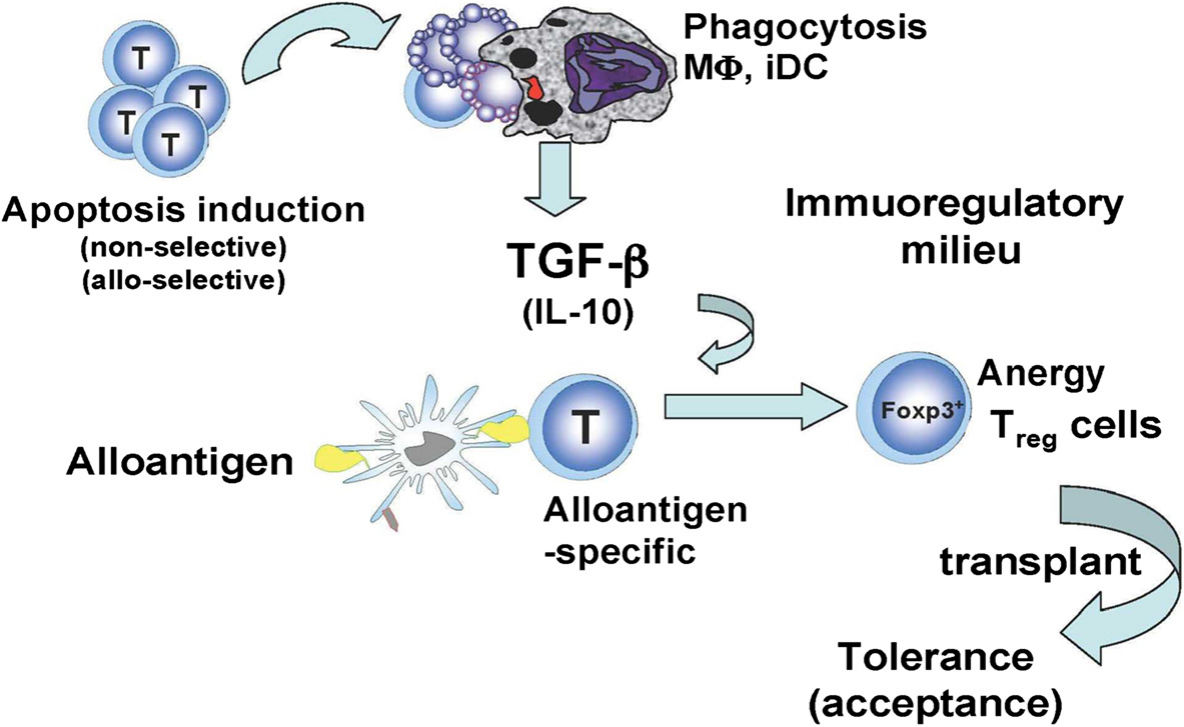
A proposed model to induce alloantigen-specific immune tolerance for transplantation. The model can be divided into three steps, i.e. induction of apoptotic cells, creation of an immunoregulatory milieu and generation of alloantigen-specific Treg cells. Once the alloantigen-specific Treg cells are generated, the allograft can be transplanted and should be accepted indefinitely. iDC: immature dendritic cells; IL-10, interleukin-10; Mo, macrophage; T, T cell; TGF-β, transforming-growth factor-β

Apoptotic cell-linked immune tolerance has implications for the prevention and inhibition of transplant rejection. We propose using allograft transplantation as the therapeutic model for demonstrating how acceptance of an allograft can be achieved by use of apoptotic T cell-mediated immune tolerance. The model can be divided into three steps: induction of apoptotic cells, creation of an immunoregulatory milieu and generation of alloantigen-specific Treg cells (Fig. 1).

#### Induction of apoptotic cells

The induction of T cell apoptosis can be divided into two pathways, non-selective and selective. The use of antibodies against T cells or certain subsets of T cells (by using for example CD3-, CD4- or CD8-specific antibodies), would induce non-selective apoptosis. Chemical or biological reagents and radioactive irradiation may also activate the non-selective pathway. The advantage of the nonselective pathway is that it can induce high numbers of apoptotic T cells. It could facilitate creating a stronger immunoregulatory microenvironment through large amounts of TGF-β production by phagocytosis of the apoptotic cells. The disadvantage of this non-selective pathway is that apoptosis of cells fighting infection and tumor cells would also be induced, although it remains unknown if this transient depletion affects the capacity of immune defense. The selective induction of apoptosis can be achieved by the depletion of particular subpopulations of T cells that respond to a specific alloantigen. A blockade of co-stimulatory molecules plus treatment with rapamycin together leading to depletion of alloantigen-responsive T cells should belong to this pathway [31, 32]. The advantage of the selective pathway is that other types of T cells are not affected, minimizing the potential side effects. The disadvantage with the selective pathway, however, is that it may be difficult to obtain sufficient numbers of apoptotic cells necessary to initiate the immunoregulatory milieu. Ideally, “apoptosis induction” should be transient and as specific as possible. It should obtain sufficient numbers of apoptotic cells but without exceeding the phagocytotic capacity of the macrophages and iDCs.

#### Creation of an immunoregulatory milieu

The uptake and digestion of apoptotic T cells by macrophages and iDCs trigger these phagocytes to produce immunoregulatory cytokines such as TGF-β and/or IL-10 and other unknown factors to create an immunoregulatory milieu. Ideally, the quantities of TGF-β and IL-10 produced should be large enough (but not overly large) to create the immunosuppressive milieu. The time period of high levels of these cytokines should not last too long, as both TGF-β and IL-10 are non-specific and execute multiple effects. It is imperative to explore and determine how long this “immunoregulatory milieu” should last.

#### Generation of alloantigen-specific Treg cells

Once the immunoregulatory milieu is established, the donor alloantigen should be introduced into the recipient to induce alloantigen-specific Treg cells. This is because it is more likely that the alloantigen specific T cells become anergic or differentiate into Treg cells in response to the donor alloantigen when in a TGF-βrich immunosuppressive microenvironment. This step is critical to transform the initial non-specific immunosuppressive steps into an alloantigenspecific immune tolerance.

After the alloantigen-specific Treg cells have been generated, the donor allograft can be transplanted and should be accepted within the host indefinitely because theoretically, the allograft could continue to provide alloantigens to maintain and expand the population of alloantigen-specific Treg cells and/or to induce new alloantigen-specific Treg cells [44]. It should be pointed out that induction and maintenance of alloantigen-specific Treg cells is the key for long-term “specific” tolerance. Although this proposed model of immune tolerance is theoretically possible, much work needs to be done before it is clinically applicable.

### Concluding remarks

A transient but sufficient number of apoptotic cells can induce a long-term immune tolerance. This process involves the production of immunoregulatory cytokines, and in particular TGF-β by the phagocytes that clear apoptotic cells. These immunoregulatory cytokines in turn help create an immunosuppressive microenvironment under which alloantigen-specific T cells are anergized or converted into alloantigen-specific Treg cells rather than differentiating into Th1, Th2 or Th17 immune inflammatory effectors. The induced alloantigen-specific Treg cells will favor the acceptance of the donor allograft from the same donor and protect the transplanted tissues from rejection. While this proposed model to induce alloantigen-specific tolerance is attractive and theoretically possible and would help us reach the “Holy Grail” of transplantation, there is clearly still a long way to go. For instance, interference by the immunosuppressive regimen (that is mandatory to avoid acute graft rejection in clinical settings) should also be considered [45]. By reinforcing our understanding of the basic cellular and molecular mechanisms of immune tolerance, we could develop more effective therapy for the patients who need transplantation.
